# Healthcare educational debt in the united states: unequal economic impact within interprofessional team members

**DOI:** 10.1186/s12909-023-04634-1

**Published:** 2023-09-14

**Authors:** Richard K. Shields, Manish Suneja, Bridget E. Shields, Josef N. Tofte, Shauna Dudley-Javoroski

**Affiliations:** 1 Department of Physical Therapy and Rehabilitation Science, Roy J. and Lucille A. Carver College of Medicine, University of Iowa, 1-252 Medical Education Building, Iowa City, IA 52242 USA; 2 Department of Internal Medicine, Carver College of Medicine, The University of Iowa, Iowa City, IA USA; 3 Department of Dermatology, School of Medicine and Public Health, University of Wisconsin, Madison, WI USA; 4 Department of Orthopedics and Rehabilitation, School of Medicine and Public Health, University of Wisconsin, Madison, WI USA

**Keywords:** Interprofessional, Debt, Education, Salary, Economics, Allied health occupations, Health workforce, Minority groups, Ethnic and racial minorities, Sexual and gender minorities

## Abstract

**Background:**

Advancing healthcare access and quality for underserved populations requires a diverse, culturally competent interprofessional workforce. However, high educational debt may influence career choice of healthcare professionals. In the United States, health professions lack insight into the maximum educational debt that can be supported by current entry-level salaries. The purpose of this interprofessional economic analysis was to examine whether average educational debt for US healthcare graduates is supportable by entry-level salaries. Additionally, the study explored whether trainees from minoritized backgrounds graduate with more educational debt than their peers in physical therapy.

**Methods:**

The study modeled maximum educational debt service ratios for 12 healthcare professions and 6 physician specialties, incorporating profession-specific estimates of entry-level salary, salary growth, national average debt, and 4 loan repayment scenarios offered by the US Department of Education Office of Student Financial Aid. Net present value (NPV) provided an estimate for lifetime “economic power” for the modeled careers. The study used a unique data source available from a single profession (physical therapy, N = 4,954) to examine whether educational debt thresholds based on the repayment model varied between minoritized groups and non-minoritized peers.

**Results:**

High salary physician specialties (e.g. obstetrics/gynecology, surgery) and professions without graduate debt (e.g. registered nurse) met debt ratio targets under any repayment plan. Professions with strong salary growth and moderate debt (e.g. physician assistant) required extended repayment plans but had high career NPV. Careers with low salary growth and high debt relative to salary (e.g. physical therapy) had career NPV at the lowest range of modeled professions. 29% of physical therapy students graduated with more debt than could be supported by entry-level salaries. Physical therapy students from minoritized groups graduated with 10–30% more debt than their non-minoritized peers.

**Conclusions:**

Graduates from most healthcare professions required extended repayment plans (higher interest) to meet debt ratio benchmarks. For several healthcare professions, low debt relative to salary protected career NPV. Students from minoritized groups incurred higher debt than their peers in physical therapy.

**Supplementary Information:**

The online version contains supplementary material available at 10.1186/s12909-023-04634-1.

## Background

As evidence accumulates for poorer health outcomes for minorities and individuals from disadvantaged socioeconomic backgrounds [1, 2], health systems are seeking ways to improve healthcare access and quality of care for underserved communities. Developing a more diverse healthcare workforce is a critical strategy for achieving this goal, reflecting both social justice imperatives [3] and opportunities for an enhanced patient experience in socio-demographically concordant patient-provider dyads [4, 5]. Students from under-represented racial, ethnic, and socioeconomic backgrounds may be more likely to practice in underserved areas and to serve underserved populations [6–8]. Thus, the recruitment and retention of healthcare trainees from these diverse backgrounds appears as priority across all healthcare disciplines [9–11]. In the United States (US), however, declining representation of these students within the educational pipeline has already been observed for several professions [9].

A healthcare student’s choice to practice in a high-need setting likely reflects a complex interplay of their demographic and socioeconomic background [7, 12], personal motivation and calling [13], exposure to underserved populations during training [14, 15], and likely, their educational debt [16–18]. Students with high educational debt may be less likely to practice in underserved areas, self-selecting instead into higher-salary specialties and geographic markets [18, 19]. In the case of medicine, five decades of rising medical student debt may have laid the foundation for the present acute shortage of primary care providers in the US [17, 20]. Rising debt in pharmacy correlates with reduced interest in post-graduate residency and hospital-based practice [21]. Physical therapy has witnessed an uptick of indebted graduates who opt for high-paying travel positions instead of their preferred practice settings [22] and who take on additional paid employment to supplement full-time wages [23]. Debt-related consequences appear to be borne unequally across the healthcare landscape, with certain professions experiencing a positive return on investment [24] and others experiencing acute pressures [19, 25–28]. Debt-reduction programs such as Title VII of the Public Health Service Act and the National Health Service Corps Scholarship Program [14] are only available to a handful of healthcare professions. Healthcare education programs themselves rarely have the institutional power to determine their own tuition [29], leaving few constraints on the costs that are ultimately transferred to students [23, 30]. Students who lack the protection of generational wealth [31], may experience more financial pressures, even if the average debt for a profession is viewed as acceptable. The critical goal of developing a diverse, culturally competent interprofessional healthcare workforce may be opposed by the student debt experienced in healthcare education.

Most healthcare professions, even those that have raised the alarm about debt, lack fundamental information for how much educational debt is “too much”, and whether that debt is evenly distributed among minoritized groups. Data sets with individual reported demographic information coupled with student debt data are not available for analysis in most healthcare professions. Accordingly, there is a need for leaders in healthcare to (1) identify maximum supportable limits for educational debt across the range of healthcare professions that comprise contemporary interprofessional healthcare teams, and (2) to strive to understand if students from racially minoritized groups have less favorable debt-service ratios than their non-minoritized peers. This second point requires that reputable data sets, with demographic and debt data, are available for analysis.

To address these gaps, we developed an economic model to estimate maximum educational debt service ratios for a broad range of healthcare careers. We modeled the net present value of these careers, creating a contextualized ranking of career “economic power” experienced by professions across the debt-to-income spectrum. In addition, using a novel dataset, we linked individual student demographic data to student debt data within one healthcare profession. Using self-reported data we explored, for the first time, if the educational debt of minoritized groups varied from that of their non-minoritized peers in physical therapy.

## Methods

### Estimating starting salary

The analysis included twelve healthcare professions, six medical specialties, and a general bachelor’s degree career (Table 1). We estimated entry-level salaries using the 25th percentile [26] of the Bureau of Labor Statistics National Occupational Employment and Wage Survey (OEWS) [32]. 10 years of wage data (2012–2021) were included in the analysis. The OEWS currently includes 19 physician specialties, but longitudinal wage data were available only for the six specialties included in the analysis. The OES began to separately report surgical subspecialties in 2019, so all surgical subspecialty data is grouped to maintain consistency with the 2012 data repository used for other specialties. Resident physician salary was obtained from the Association of American Medical Colleges Resident Survey [33].

**Table 1:** NPV model inputs, with physician specialties grouped in the lower rank of rows

						Monthly Payment ($)						Debt Ratio (%)^a^					Total Repaid^b^				
	2021 salary ($)	10-yr CAGR (%)	Undergrad debt ($)	Grad debt ($)	Max Debt Ratio (%)	Stand.	Ext.	Ext Grad start	Ext Grad end	PAYE & IBR start	PAYE & IBR end	Stand.	Ext.	Ext Grad start	Ext Grad end	PAYE & IBR start/end	Stand.	Ext.	Ext Grad	PAYE & IBR	PAYE & IBR Forgiveness ($)
Audiology	71,390	2.40	20,566	140,349	14	1720	956	688	1549	425	685	***28.9***	***16.1***	11.6	14.4	7.1/7.3	206,383	286,927	313,753	130,927	195,428
Bachelor’s Degree	47,164	2.15	29,096		11	301				223	288	7.7				5.7/5.6	36,101			38,630	
Chiropractic	50,470	0.92	20,566	115,668	12	1435	786	559	1275	251	242	***34.1***	***18.7***	***13.3***	***24.1***	6.0/4.8	172,212	235,720	256,675	59,638	208,214
Dentistry^c^	116,990	1.17	16,700	284,900	16	3232	1803	1305	2903	805	968	***33.2***	***18.5***	13.4	***22.3***	8.3/7.9	387,866	540,891	591,293	212,473	403,622
Genetic Counselor^d^	76,300	2.91	20,566	58,415	15	840	464	330	761	466	773	13.2	7.3	5.2	5.8	7.3/8.6	100,796	139,341	152,449	121,804	0
Nurse Practitioner^e^	99,540	2.41	20,566	55,000	16	784	421	294	691	660	802	9.5	5.1	3.5	4.6	8.0/7.3	94,031	126,440	137,230	96,790	0
Occupational Therapy	75,710	1.93	20,566	58,415	15	840	464	330	761	461	653	13.3	7.4	5.2	7.5	7.3/7.1	100,796	139,341	152,449	127,818	0
Optometry	96,230	2.51	11,324	147,524	16	1692	938	675	1513	632	1016	***21.1***	11.7	8.4	10.2	7.9/7.7	203,077	281,404	307,149	194,325	116,629
Pharmacy	121,070	1.60	20,566	173,561	17	2057	1133	812	1832	839	1073	***20.4***	11.2	8.0	12.2	8.3.7.7	246,818	340,002	370,645	228,467	144,778
Physical Therapy	77,750	1.51	16,804	82,788	15	1063	590	423	959	478	594	***16.4***	9.1	6.5	10.2	7.4/6.8	127,537	177,003	193,585	128,295	58,708
Physician Assistant	99,880	2.42	30,000	105,000	16	1438	796	568	1301	662	1065	***17.3***	9.6	6.8	8.6	8.0/7.9	172,535	238,905	261,345	203,649	35,064
Radiation Therapy	75,490	1.77	29,096	21,436	15	531	290	199	488	459	531	8.4	4.6	3.2	5.0	7.3/7.0	63,726	86,882	95,187	65,694	0
Registered Nurse	61,790	1.42	29,096		13	301				345	375	5.8				6.7/6.5	36,101			34,793	
Internal Medicine^f,h^	122,140	0.50	28,000	200,000	17	2437	1356	975	2195	848	853	***23.9***	13.3	9.6	***19.0***	8.3/7.6	292,478	406,709	444,731	217,449	236,421
Psychiatry^h^	128,380	0.64	28,000	200,000	17	2437	1356	975	2195	900	967	***22.8***	12.7	9.1	***17.5***	8.4/8.6	292,478	406,709	444,731	224,468	238,512
Pediatrics^h^	129,410	0.50	28,000	200,000	17	2437	1356	975	2195	909	920	***22.6***	12.6	9.0	***18.0***	8.4/8.2	292,478	406,709	444,731	220,054	243,041
Family Medicine^h^	143,830	1.06	28,000	200,000	17	2437	1356	975	2195	1029	1182	***20.3***	11.3	8.1	14.1	8.6/8.0	292,478	406,709	444,731	265,208	182,633
Obstetrics/Gynecology^h^	198,290	1.80	28,000	200,000	18	2437	1356	975	2195	1483	1966	14.7	8.2	5.9	8.5	9.0/10.3	292,478	406,709	444,731	352,452	0
Surgery^g,h^	207,720	1.31	28,000	200,000	18	2437	1356	975	2195	1561	1864	14.1	7.8	5.6	9.2	9.0/9.7	292,478	406,709	444,731	347,513	0

^a^Italicized/bolded text denotes repayment plans exceeding the profession’s recommended debt service ratio for entry-level salary. ^b^Professions entered repayment at post-baccalaureate year 1 (bachelor’s degree, registered nurse), year 3 (genetic counseling, radiation therapy), year 4 (occupational therapy, physical therapy, physician assistant), or year 5 (audiology, chiropractic, dentistry, medicine, nurse practitioner, optometry, pharmacy). Debt forgiven under income-contingent plans (PAYE and IBR) is taxable income. ^c^Dentistry salary and CAGR used 2020 data (9-year CAGR) because the 2021 salary estimate is an outlier. ^d^Genetic counselor 10-yr CAGR (7.16) is not likely sustainable: substituted CAGR for all professions/economic sectors. ^e^The modeled nurse practitioner career assumed a 4-year Doctor of Nursing Practice degree, but national debt estimates include nurse practitioners entering the profession with 2–3 year Master’s degree training. ^f^Internal medicine 10-yr CAGR (-1.16) reflects 5 wage reversals in 10 years and is unsuited to modeling. Substituted next-closest physician specialty (Pediatrics). ^g^Surgery salary and CAGR used 2019 data (8-year CAGR) because later data exclude surgical subspecialties. ^h^For all physician specialties, resident physician salary, CAGR, and total tax rate were input for the first three years of loan repayment regardless of eventual specialty choice. At repayment year 4 the model incorporated specialty-specific values for salary (adjusted via CAGR for residency years), salary CAGR, and tax rate

For each profession/specialty, we calculated the compound annual growth rate (CAGR) of entry-level salary using the formula: (((2021 salary/2012 salary)^(1/10 years))-1) x 100. CAGR is a smoothed, annualized estimate of change across a defined time that limits the influence of wage volatility in individual years. We calculated compound annual inflation rate (CAIR) using the same method and January-to-January inflation data from the Consumer Price Index [34].

### Estimating student debt and repayment

Data sources for national average student debt included U.S. healthcare professions’ national professional organizations, government databases, education research foundations, and peer-reviewed papers (Please see Additional File 1) [35–43]. We estimated annual student loan payments using the US Department of Education Student Loan Simulator [44]. The interest rate for undergraduate debt was modelled using the mean of historic interest rates for Direct Subsidized Loans for 2017–2020 (4.45%) [45]. The interest rate for graduate debt was modelled using the mean of historic interest rates for Direct Unsubsidized Loans for 2020–2022 (5.27%) [45]. Repayment plans were simulated for a single borrower with entry-level salary and salary CAGR as described in Table 1. The analysis focused on four repayment plans representing the breadth of repayment terms available to most borrowers: Standard (fixed payments, 10 years), Extended (fixed payments, 20 years), Extended-Graduated (escalating payments, 25 years) and two income-contingent plans (Pay As You Earn (PAYE) and Income Based Repayment (IBR)). Debt service ratios for the end of the Extended Graduated plan were calculated using CAGR-projected future salary. Repayment under PAYE and IBR is linked to salary growth over time (CAGR) and is capped at 10% of discretionary income (defined as salary exceeding 150% of the poverty line). At the end of 20 years, any remaining debt under these plans is forgiven, with the forgiven balance reported as taxable income. Public Service Loan Forgiveness (PSLF) program repayment was not modeled in this analysis. Repayment scenarios for PSLF would be identical in most cases to PAYE or IBR, but any amount forgiven after 20 years would not be considered taxable income. The analysis assumed that the CARES Act payment and interest moratorium would end as scheduled in 2023 and that no federally initiated universal loan forgiveness would occur.

### Estimating debt service ratios

In 2006 the Student Debt Project and the College Board developed income-linked debt service ratio benchmarks that reflect the maximum percentage of a borrower’s discretionary income that should be used for student loan repayment [46]. By linking maximum debt service ratio to discretionary income (funds left over after payment of essentials), this model acknowledges that higher-earning professions such as physicians can generally devote a higher percentage of overall income to debt repayment than lower-paying professions, for whom discretionary income is lower. File 2 describes how the 2006 benchmarks were validated to reflect 2022 financial conditions [46, 47]. This conversion yielded a debt service ratio for each profession that signified maximum salary-linked capacity to repay educational debt (Table 1).

### Net present value

Net present value (NPV) is an economic modeling approach used to estimate long-term “economic power” for careers [48]. NPV captures the monetary difference between a benefit to be gained (e.g. a healthcare career) and the cost required to obtain the benefit (e.g. educational debt required for entry to a healthcare career). It may also model “opportunity cost” for factors such as foregone income from higher-paying careers and earlier initiation of wage-earning for careers with shorter educational periods. In all cases, the monetary value of each future benefit or cost (C) is expressed in current monetary terms (“present value”: PV) by applying a discount rate (r) over (t) years in the future: PV = C/(1 + r)^t^.

Selection of a discount rate is one of the most important factors in development of a NPV model because it exerts a strong influence on the final modeled PV for a career, which may skew cost-benefit decisions toward or against a modeled career. An ideal discount rate would closely approximate future economic conditions for an industry, e.g., healthcare. However, the fidelity of a modeled discount rate to future economic conditions cannot be determined *a priori*. Previous healthcare salary NPV models have used a range of discount rates, with 5% being widely selected [26, 49–51]. A panel of economic experts proposed 3% as a discount rate for healthcare costs [52], and this less conservative rate has also been applied to healthcare salaries [53]. The present study modeled both 3% (less conservative) and 5% (more conservative) discount rates for healthcare salary PV.

Career NPV was modeled as (PV[after-tax career earnings] – PV[cost of student loan repayment] – PV[opportunity cost of an alternate career]). Federal tax was estimated using the 2022 tax Table [54] and state tax was modeled as the mean for all states and the District of Columbia (5%) [55]. Retirement at age 65 was assumed for all professions. Student loans were assumed to encapsulate the economic cost of obtaining a healthcare education. Student loan repayment was modeled using the repayment plan that yielded the most rapid payoff (and thus the lowest accumulated interest), but that did not exceed the recommended debt service ratio for that profession (Table 1). Close examination of Table 1 shows the Debt Ratio (%) across the 5 repayment plan methods. The italicized/bolded text depicts debt ratios that exceed the recommended repayment threshold (> 15% of discretionary income), based on the economic model. As an example, surgery and obstetrics/gynecology show no italicized/bolded debt ratios, indicating that based on the economic model, all repayment plans fall below the recommended 15% of discretionary income. Even with an adjustment in the length of residency to 5 years rather than 3 years, these two medical specialties fell below the recommended 15% of discretionary income. Table 2 shows an example of the effect of varying degrees of total debt on the repayment plan that would be recommended in physical therapy. A debt > $150,000 begins to limit the number of plans that are available, if the goal is to pay less than the recommended 15% of discretionary income.

**Table 2:** Loan repayment scenarios for physical therapy graduates

	Debt ($)^b^			Monthly Payment ($)						Debt Ratio^c^					Total Repaid / Forgiven ($)^d^				
Total Debt ($)^a^	% of Cohort†	Undergrad debt	PT school debt	Standard	Extended	Ext Grad (start)	Ext Grad (end)	PAYE/ IBR (start)	PAYE/ IBR (end)	Standard	Extended	Ext Grad (start)	Ext Grad (end)	PAYE & IBR (start/end)	Standard	Extended	Ext Grad	PAYE/ IBR	PAYE/IBR Forgiveness
75,000^e^	20.5	16,804	58,196	799	442	315	723	478	587	12.3	6.8	4.9	7.7	7.4 / 6.8	95,846	132,705	143,514	118,016	$0
99,592^f^	13.6	16,804	82,788	1063	590	423	959	478	594	***16.4***	9.1	6.5	10.2	7.4 / 6.8	127,537	177,003	193,585	128,295	58,708
125,000	10.0	16,804	108,196	1336	743	534	1204	478	594	***20.6***	11.5	8.2	12.8	7.4 / 6.8	160,280	222,770	243,605	128,295	125,144
150,000	10.3	16,804	133,196	1604	893	643	1444	478	594	***24.8***	*13.8*	9.9	***15.3***	7.4 / 6.8	192,497	267,802	292,823	128,295	176,721
175,000	8.0	16,804	158,196	1873	1,043	752	1685	478	594	***28.9***	***16.1***	11.6	***17.9***	7.4 / 6.8	224,714	312,834	342,042	128,295	227,785
200,000	7.3	16,804	183,196	2141	1,193	861	1925	478	594	***33.0***	***18.4***	*13.3*	***20.4***	7.4 / 6.8	256,931	357,866	391,257	128,295	239,473
225,000	4.3	16,804	208,196	2410	1,343	970	2166	478	594	***37.2***	***20.7***	***15.0***	***23.0***	7.4 / 6.8	289,148	402,898	440,475	128,295	330,110

^a^Debt < $75,000 was not modeled because payments would meet the profession’s 15% debt ratio benchmark under any repayment plan. ^b^The model assumes undergraduate debt equal to the mean for the PT-GQ cohort ($16,804). ^c^Repayment plans approaching the recommended debt service ratio for physical therapist starting salaries are shown in italicized text. Repayment plans exceeding this limit are shown in bolded/italicized text. ^d^Debt forgiven under PAYE and IBR (income-contingent) plans is taxable income. ^e^The $75,000 tier is cumulative from $1 to $75,000. All other tiers are cumulative from the preceding tier. ^f^$99,592 = mean total debt for the Benchmarking in PT Education study national sample

### Physical therapy NPV for minoritized groups

Individual data for educational debt of students from minoritized groups for healthcare professions is a challenge to obtain. Because we are the principal investigators of the national Benchmarking in Physical Therapy Education study, we modeled physical therapy (physical therapy; PT) using data from this ongoing national study (N = 4,954) [56–58]. Collection of this data was approved by the University of Iowa Human Subjects Institutional Review Board and offered a unique opportunity to examine the impact of student debt on minoritized groups. Accordingly, we examined total educational debt (undergraduate plus graduate debt) for PT students from three minoritized groups: racial and ethnic minority (REM: any student with a non-white or Latino/a/x identity, including biracial and multiracial); sexual and gender minority (SGM: any student identifying as non-heterosexual and/or non-cisgender); and socioeconomic disadvantage (SED: any first-generation college student and/or self-identification with a socioeconomic disadvantaged background). We compared total educational debt and the percent of each minoritized group that exceeded the $150,000 threshold that limited repayment options (Table 2) and tested via one-way ANOVA. Total educational debt was compared among four race/ethnicity groups (each with N > 150: Asian, Black/African American, Latino/a/x, White) via Welch’s t-test with Benjamini-Hochberg false discovery rate (FDR) adjustment for multiple comparisons. Chi-squared tests were used to compare the proportion of students with debt exceeding the maximum supportable benchmark ($150,000 [26]) within each minoritized/non-minoritized dyad. Fisher’s exact tests with FDR adjustment were used to examine proportions of students with debt >$150,000 across the four analyzed race/ethnicity groups. Significance for all statistical tests was set to p < 0.05.

## Results

Table 1 displays the maximum salary-linked debt service ratio for each modeled profession/specialty. Given reported mean levels of educational debt, 6 professions and two physician specialties could use the Standard repayment plan without exceeding the recommended debt service ratio. These include a general bachelor’s degree career, registered nurse, and radiation therapy, which requires a certification program but not a graduate degree. The three remaining professions (genetic counseling, occupational therapy, nurse practitioner) currently can be pursued with Masters’ degree training. High salaries for obstetrics and gynecology and for surgery would permit these specialties to use the Standard repayment plan.

Repayment options narrowed for other professions/specialties, with most requiring the Extended repayment plan (20 years of fixed payments) to remain below the maximum debt ratio benchmark (please see italicized/bolded debt ratio % in Table 1). These careers would also have the option of using the Extended Graduated plan, which offers escalating payments over 25 years. Low salary growth for dentistry, internal medicine, psychiatry, and pediatrics would cause the Extended Graduated plan to exceed the maximum debt ratio near the end of the repayment term, potentially exposing mid-career professionals to financial difficulty.

Chiropractic practitioners with the reported entry-level salary and educational debt would not meet the maximum debt ratio under any conventional plan (italicized/bolded debt ratio % in Table 1). The PAYE and IBR income-contingent plans would cap loan payments at 10% of discretionary income, enabling this profession to meet their debt ratio benchmark. $208,214 in debt would be forgiven after 20 years; however, this would be reported as taxable income in the year of loan forgiveness. Income-contingent plans for several other professions would likewise create large tax obligations due to forgiven educational debt.

**Fig. 1:**
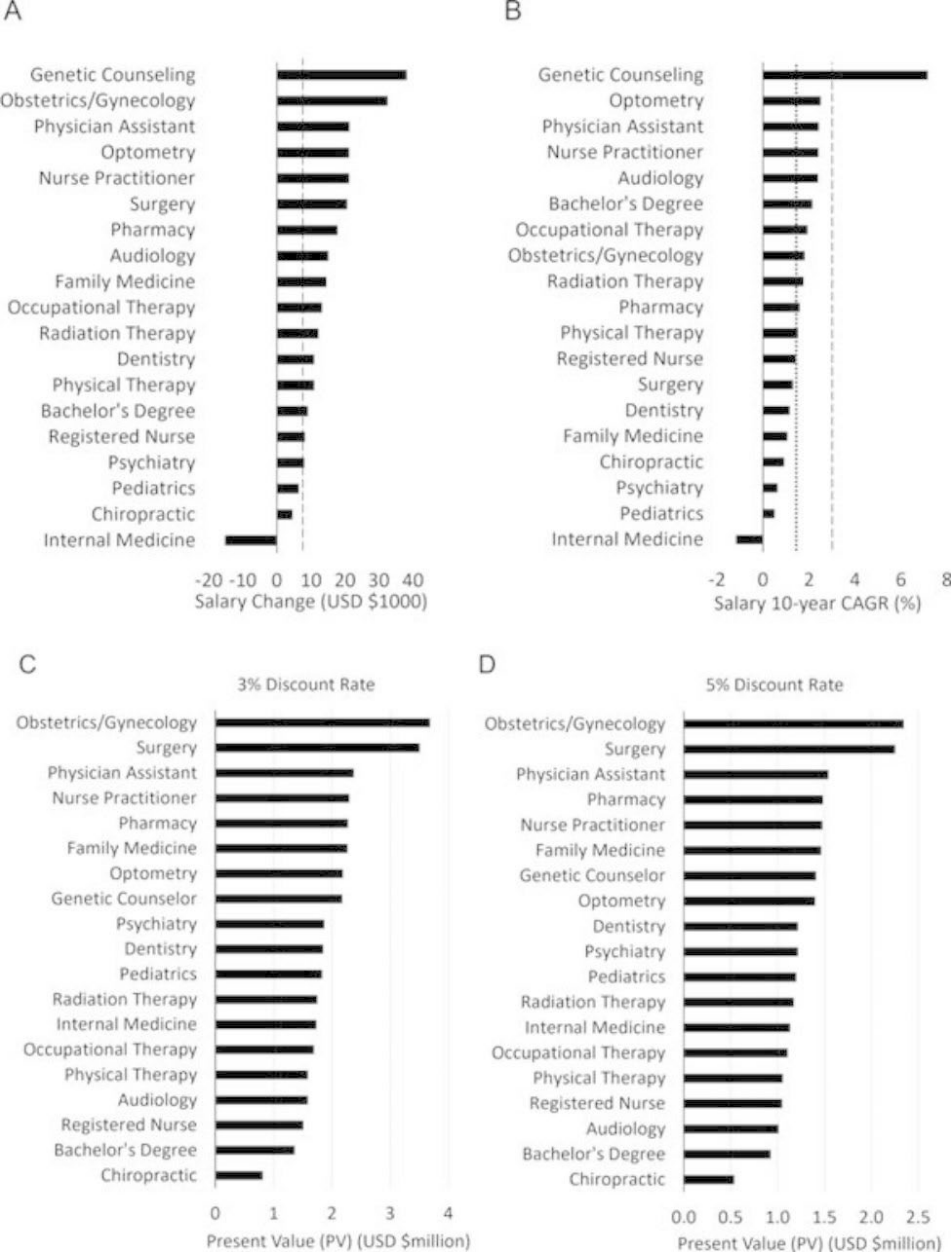
Salary change and present value (PV) for healthcare professions. **A**) Entry-level salary change (2012–2021): the dashed line depicts all professions in all economic sectors ($7,470). **B**) 10-year compound annual growth rate (CAGR) of entry-level salary: the dashed line depicts all professions in all economic sectors (2.91) and the dotted line represents the compound annual inflation rate (CAIR: 1.41). **C** and **D**) Present value (PV) analysis of healthcare professions, modeled with less-conservative (**C**) and more-conservative (**D**) discount rates

Figure 1 depicts 10-year (2012–2021) entry-level salary change for modeled professions. While healthcare salary growth in dollars generally exceeded the mean for all economic sectors (Fig. 1A), the rate of salary growth (CAGR) for most healthcare professions did not keep pace with other economic sectors (Fig. 1B). The notable exception was genetic counseling, which experienced rapid salary growth in the modeled period. Salary growth for 7 of 19 modeled healthcare professions failed to match the rate of inflation over the study period (Fig. 1B), including 5 of 6 modeled physician specialties. Physical therapist salary growth exceeded inflation by 0.1%.

Figure 1 illustrates modeled PV for healthcare careers using a less conservative (3%, Fig. 1C) and more conservative (5%, Fig. 1D) discount rate. At each rate, PV for careers in obstetrics and gynecology and surgery were substantially higher than other modeled professions. The strong PV for physician assistant, pharmacy, and nurse practitioner careers reflects the combined influence of moderate to high starting salary, robust salary growth, and in the case of physician assistants and nurse practitioners, low student debt (Table 1). In contrast, PV for chiropractic was the lowest of all modeled professions and did not meet the PV of a bachelor’s degree.

Table 2 illustrates repayment scenarios for PT students across various Total Debt projections. This table offers a more detailed example, in physical therapy, as to where the threshold for total debt influences the repayment plan that meets the recommended 15% of discretionary income. Because we have “actual” data for physical therapy, we knew that the Mean total educational debt for the sample was $99,592, comprising $16,804 in undergraduate debt and $82,788 in PT school debt. Students with this level of debt would require the Extended (20 year) repayment plan to meet the 15% debt ratio benchmark. However, at $150,000 in debt, viable repayment options dwindle for PT students: the Extended plan approaches the upper limit of the acceptable debt service ratio, and the Extended-Graduated plan would not offer a suitable debt ratio near the end of the repayment term. 28.9% of graduating PT students in the PT-GQ cohort reported debt at or above this level. At $200,000 in debt, the Extended-Graduated plan would approach the upper limit of the acceptable debt service ratio, leaving income-contingent plans as the remaining options. Physical therapists with $200,000 debt who receive loan forgiveness under these plans would report an additional $239,000 in taxable income in the year of loan forgiveness. Based on these findings, we sought to establish the percent of minoritized groups that exceed the $150,000 debt threshold, indicating that repayment models begin to erode (see below), and based on actual self-reported data in physical therapy.

**Fig. 2:**
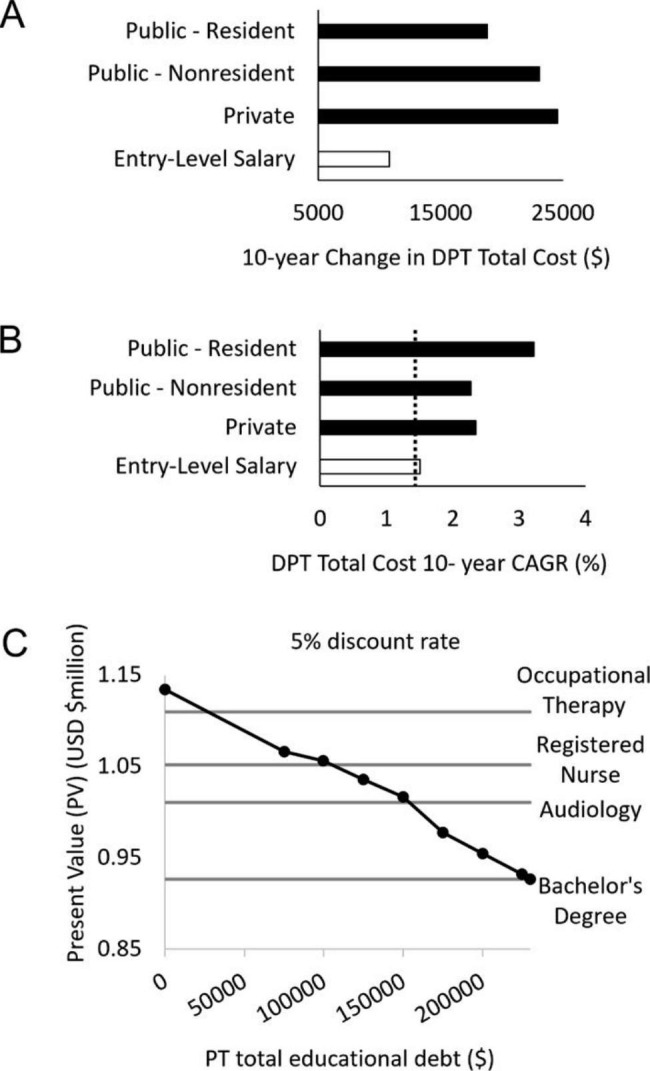
Doctor of physical therapy (DPT) degree cost and PT career present value (PV). **A**) 10-year trends in total cost of the DPT degree, compared to growth in entry-level PT salaries. **B**) 10-year compound annual growth rate (CAGR) for total cost of the DPT degree versus entry-level PT salaries. The dotted line represents the compound annual inflation rate (1.41). **C**) Present value (PV) of a career in PT at several tiers of total educational debt, modeled at a 5% discount rate. Horizontal lines indicate when the PV of a career in PT falls below other healthcare professions, culminating with no PV difference from a bachelor’s degree with $230,000 in total educational debt

Figure 2 illustrates 2012–2022 growth in cost of the Doctor of Physical Therapy (DPT) degree [59] versus growth in entry-level salaries, both in terms of dollars (Fig. 2A) and CAGR (Fig. 2B). Entry-level PT salaries outpaced inflation by 0.1%, whereas the cost of the DPT degree exceeded inflation (1.41%) by 0.9–1.8%. This represents 1.5 to 2.1-fold faster growth in the cost of the DPT degree than the rate of inflation or entry-level salaries.

Figure 2 C illustrates the PV of a career in physical therapy for graduates with debt across the range of values observed with actual reported numbers from the PT-GQ cohort. Students with $0 debt (16.7% of the sample) would experience a career PV exceeding occupational therapy. 49.2% of the sample had debt exceeding the profession’s mean of $99,592: these individuals would experience a lower career PV than a registered nurse. At total debt above $150,000 (28.9% of the sample), the PV of a career in physical therapy falls below Audiology, leaving chiropractic as the final healthcare profession with a lower PV (data not shown). At total debt above $230,000 (8.6% of the sample), the PV of a career in physical therapy no longer exceeds that of a Bachelor’s degree.

**Fig. 3:**
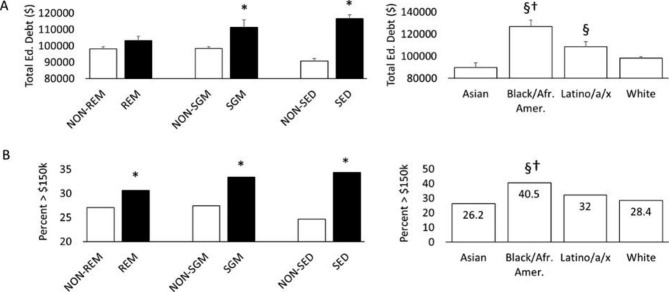
Physical therapist student debt for minoritized groups. Racial and ethnic minority (REM), sexual and gender minority (SGM), and socioeconomic disadvantage (SED). All values include individuals with $0 scholarship / debt. * = Significantly different from non-minoritized group; § = significantly different vs. Asian; † = significantly different vs. White

Figure 3 illustrates differences in total educational debt for PT students from minoritized and non-minoritized groups. Sexual and gender minority (SGM) and socioeconomically disadvantaged (SED) students reported 13.2% and 28.3% higher total debt, respectively, than their non-minoritized counterparts (both p < 0.05)(Fig. 3A). Latino/a/x students and Black/African American students reported 21.2% and 41.2% higher total debt, respectively, than Asian students (both p < 0.05). In addition, Black/African American students reported 28.9% higher debt than White students (p < 0.05). Figure 3B depicts differences in the proportion of students from minoritized and non-minoritized groups with debt exceeding the maximum $150,000 recommended limit. The proportion of REM, SGM, and SED students with total debt exceeding this benchmark was 3.6%, 5.9%, and 9.7% higher than their non-minoritized counterparts, respectively (all p < 0.05). As calculated by our methodology, the proportion of Black/African American students with total debt at or exceeding $150,000 was 40.5%. This proportion was 12.1% and 14.3% higher than White and Asian students, respectively (both p < 0.05) and 11.7% higher than the average for the PT-GQ sample as a whole.

## Discussion

The study presents maximum debt ratio benchmarks for a range of healthcare professions that require baccalaureate through post-graduate education. The benchmarks can assist healthcare professions to address fundamental questions about how much debt can be supportable by standard entry-level salaries and to evaluate circumstances where salaries differ from the national average (e.g., certain practice settings or geographic regions). Moreover, these debt ratio benchmarks can assist student borrowers to evaluate their own plans to take on educational debt, especially in cases where individual starting salaries deviate from nationally reported averages. This guidance may be especially valuable to students who intend to practice in lower-paying, underserved markets that offer lower than average pay. Taken together, these findings highlight specialties and individual health professions that may face challenging financial futures.

### Career economic power for healthcare professions and academic institutions

Using published estimates of entry-level salary, salary growth, and average educational debt, we evaluated career “economic power” of healthcare professions in terms of NPV, capturing the economic benefit of a career versus the cost (including opportunity cost) of healthcare education. At national-average salary and debt levels, highly paid physician specialties (obstetrics & gynecology, surgery) were unlikely to experience restricted loan repayment options or an excessive debt service ratio (Table 1). Excessive debt service ratios did not emerge for these two specialties, even when the model included two additional years of residency beyond what was modeled for all other specialties. Professions without graduate debt (bachelor’s degree, radiation therapy, registered nurse) met debt ratio limits under any repayment plan, as did several professions with moderate debt relative to salary (genetic counselor, nurse practitioner, occupational therapy) (Table 1). The remaining healthcare professions experienced narrower repayment options that required longer repayment durations and higher total interest. For these professions, consideration of NPV may provide insight into the cost-benefit balance of educational debt. However, it is imperative that healthcare professionals weigh their own individual situations as many other factors will influence Debt Ratios (%). For example, new spousal support, spousal debt, bonus payment opportunities, debt forgiveness programs, private vs. public employment, family generational wealth support/loss that emerges later during a professional career will all impact the rate that one can repay debt. These factors, and others, are incorporated into this economic model. By understanding the impact of salary, salary growth rate, educational debt, and recommended repayment thresholds (15% of discretionary income), healthcare professionals may have greater insights about their economic commitment when entering a given healthcare profession as outlined in Table 1.

Consistent with another recent analysis [24], the narrowed loan repayment options experienced by physician assistants did not diminish career NPV (Table 1). At both modeled discount rates, physician assistant career PV was second only to the highest-paid physician specialties. Thus, the career economic outlook is positive for physician assistants, so long as salary and debt approximate national averages. This is in contrast to the outlook for dentistry, a higher-paid profession, which has moderately weak salary CAGR and higher debt than all other modeled professions. NPV comparison between physician assistants and dentistry helps illustrate the advantages that can be gained when professions with moderate salary keep educational debt low.

Four professions with similar, moderate entry-level salary ($75-$78,000) illustrate how the rate of salary growth and total educational debt affect the lifetime economic power of a career (Table 1). Strong salary CAGR and moderate total debt (<$80,000) provided genetic counseling with a PV that exceeded higher-paid professions such as dentistry, psychiatry, pediatrics, and internal medicine. Radiation therapy and occupational therapy (OT), with lower salary CAGR and/or greater debt, experienced a lower career PV despite having a similar salary to genetic counseling. All of the aforementioned professions require a post-baccalaureate certificate (radiation therapy) or a Master’s degree (genetic counseling, OT). For PT, a profession requiring doctoral-level training, low salary growth and high total debt in relation to salary ($99,592) yielded a career PV just above a Bachelor’s-trained registered nurse (5% discount rate, Fig. 1D). We suggest that rapid growth in the cost of the DPT degree without commensurate growth in entry-level PT salaries, as depicted in Fig. 2, is the key assessment of this phenomenon. Optimistic-sounding government projections about jobs growth (“much faster than the average for all occupations” [60]) may not adequately communicate the economic reality experienced by many healthcare providers, including PTs. Other professions have likewise raised concerns about declining return on investment for healthcare education [61], proposing solutions such as capping tuition, increasing scholarships, and decoupling the cost of healthcare education from the costs of institutions’ research and clinical missions [20]. Improving entry-level salary growth is an equally-important remediating strategy, but for some professions this factor is strongly limited by low reimbursement rates for services [62, 63].

Confirming previous work [26], the present study supported that educational debt of approximately $150,000 is likely to be the maximum that can be supported by current entry-level PT salaries; a debt encumbrance reported by ~ 30% of all graduates (28% whites; 40% Black/African Americans) (Table 2). Importantly, highly indebted graduates who must use income-contingent repayment plans may face a scenario known as a “student loan tax bomb”, in which forgiven debt triggers a substantial tax obligation in the year of loan forgiveness. Graduates from any profession who use income driven repayment plans should estimate this tax obligation early during repayment and plan accordingly. No data are currently available for any healthcare field on the proportion of graduates who must use income driven repayment plans. General perusal of healthcare student blogs and financial planning web resources provides a troubling impression that this is not a rare situation.

### Educational debt of minoritized groups

Recent findings indicate that representation of Black/African American students within the US healthcare educational pipeline has declined for 4 of the professions included in this analysis (physician assistant, physical therapist, occupational therapist, registered nurse) [9, 64]. Without the successful recruitment and retention of students from minoritized backgrounds, healthcare professions will struggle to meet the needs of a more racially and socioeconomically diverse US population. Consistent with trends observed for medical students [65] and more broadly across higher education [66], the present analysis confirmed that students from backgrounds that are under-represented in physical therapy [67] (Black/African American, Latino/a/x, and SED) graduated with higher educational debt than their non-minoritized peers. A substantial proportion (40.5%) of Black/African American students reported a level of debt that placed their career PV below most of the modeled professions. Regardless of racial/ethnic background, students from socioeconomically disadvantaged backgrounds (SED) incurred 19.6% ($18,375) more PT school debt than their non-SED peers. Medicine has observed a dramatic reduction in trainees from middle and lower socioeconomic backgrounds since the dawn of the student loan era [19] and now PT may be poised for a similar shift. Now that a career in PT requires doctoral training, need-based scholarship programs do not appear to adequately offset the limited family resources for education [31] available to SED individuals. A novel finding of this study was that sexual and gender minority (SGM) students also incurred higher educational debt (13.2%; $12,947) than their peers, consistent with reports from the general undergraduate population [68] but not previously observed in other healthcare professions [69]. Understanding how debt impacts those belonging to one, two, or more minoritized groups (Black/African American, Sexual Gender Minority, Socioeconomic Disadvantage) is important, but was not feasible given the limited sample sizes once the groups become stratified. Future studies are planned to better understand the intersectionality associated with people who fall into multiple minoritized categories.

### Study limitations

The strength of a model is that it is governed by consistent data but still offers meaningful information. The NPV model has inherent limitations that warrant comment. First, several physician specialties (e.g. cardiology, dermatology, orthopedic surgery) could not be modeled because longitudinal (10-year) specialty-specific salary data are not yet available from the Bureau of Labor Statistics. Government-reported salary for physician specialties does not reflect additional (often substantial) income sources such as practice financial performance and productivity-based bonus payments [70]. The model assumed 3 years for physician residency to promote consistency. When we assessed this factor, specialties with longer residency durations were still associated with more discretionary income because of higher wages (surgery, obstetrics, and gynecology) and had a negligible effect on the repayment plans. National average student debt used in the model reflected best-available data sources, but direct student survey data and/or contemporary estimates were not available for several professions (Please see File 1). The lack of publicly available individual demographic data along with self-reports of debt precluded us from examining whether other professions’ minoritized students incurred greater educational debt than non-minoritized peers. Additional research is needed to clearly understand the educational debt among people with who fall into multiple minoritized groups.

## Conclusions

Updated debt service ratio benchmarks, together with national estimates of entry-level salaries, yielded benchmarks for maximum educational debt for a wide range of healthcare professions. Modeling loan repayment scenarios and career net present value offered insights into the economic costs and benefits that “typical” graduates in these professions may experience. Using this information, healthcare trainees may make more informed decisions about how much educational debt is desirable to attain the economic and intangible benefits of a career in healthcare (Table 2). This approach may be especially useful for students from minoritized backgrounds, who may require more debt in order to gain access to healthcare professions, and for students who wish to pursue socially motivated careers, who may earn lower-than-average salaries. The present study offers a useful blueprint for healthcare professions to examine educational debt for their own trainees, particularly those from minoritized groups. For professions with problematic debt, accreditation standards, curricular adjustments, tuition-reduction efforts, and salary advocacy by professional organizations may all positively affect educational return on investment. To meet society’s need for diverse, culturally competent interprofessional teams, all professions must engage together with this complex issue.

### Electronic supplementary material

Below is the link to the electronic supplementary material.

Supplementary Material 1

Supplementary Material 2

## Data Availability

The datasets generated and/or analyzed during the current study are not publicly available due to confidentiality requirements of the national benchmarking study for physical therapist education but are available from the corresponding author on reasonable request.

## Abbreviations

CAGR: Compound annual growth rate
CAIR: Compound annual inflation rate
DPT: Doctor of Physical Therapy
FDR: False discovery rate
IBR: Income Based Repayment
NPV: Net present value
OEWS: Occupational Employment and Wage Survey
OT: Occupational therapy
PAYE: Pay As You Earn
PSLF: Public Service Loan Forgiveness
PT: Physical therapy
PV: Present value
REM: Racial and ethnic minority
SED: Socioeconomic disadvantaged
SGM: Sexual and gender minority
US: United States
