# Supplementary material for: Healthcare educational debt in the united states: unequal economic impact within interprofessional team members
Source: BMC Med Educ. 2023 Sep 14;23:666. doi: 10.1186/s12909-023-04634-1 (PMC10503048; doi:10.1186/s12909-023-04634-1)
Supplement: Supplementary file 1 — Supplementary Material 1 [file 12909_2023_4634_MOESM1_ESM.docx]

**Supplement 2. Revised Debt Ratio Tiers**

Conversion of 2006 debt service ratio tiers from Baum and Schwartz / The College Board (2006) for use with 2021 income tiers.

| **2021 Income Tier** | **Discretionary Income^a^** | **Annual Payment (20% of discretionary income)** | **Payment / Total Income^b^** | **Modeled Profession** |
| --- | --- | --- | --- | --- |
| $15,000 | $0 | $0 | 0% |  |
| $20,000 | $0 | $0 | 0% |  |
| $25,000 | $4,615 | $923 | 4% |  |
| $30,000 | $9,615 | $1,923 | 6% |  |
| $35,000 | $14,615 | $2,923 | 8% |  |
| $40,000 | $19,615 | $3,923 | 10% |  |
| $45,000 | $24,615 | $4,923 | 11% | Bachelor's Degree |
| $50,000 | $29,615 | $5,923 | 12% | Chiropractic |
| $55,000 | $34,615 | $6,923 | 13% |  |
| $60,000 | $39,615 | $7,923 | 13% | Registered Nurse |
| $65,000 | $44,615 | $8,923 | 14% |  |
| $70,000 | $49,615 | $9,923 | 14% | Audiology |
| $75,000 | $54,615 | $10,923 | 15% | Genetic Counseling, Occupational Therapy, Physical Therapy, Radiation Therapy |
| $80,000 | $59,615 | $11,923 | 15% |  |
| $85,000 | $64,615 | $12,923 | 15% |  |
| $90,000 | $69,615 | $13,923 | 15% |  |
| $95,000 | $74,615 | $14,923 | 16% | Optometry |
| $100,000 | $79,615 | $15,923 | 16% | Nurse Practitioner^c^, Physician Assistant^c^ |
| $105,000 | $84,615 | $16,923 | 16% |  |
| $110,000 | $89,615 | $17,923 | 16% |  |
| $115,000 | $94,615 | $18,923 | 16% | Dentistry |
| $120,000 | $99,615 | $19,923 | 17% | Internal Medicine, Pharmacy |
| $125,000 | $104,615 | $20,923 | 17% | Psychiatry, Pediatrics |
| $130,000 | $109,615 | $21,923 | 17% |  |
| $135,000 | $114,615 | $22,923 | 17% |  |
| $140,000 | $119,615 | $23,923 | 17% | Family Medicine |
| $145,000 | $124,615 | $24,923 | 17% |  |
|  |  |  |  |  |
| $195,000 | $174,615 | $34,923 | 18% | Obstetrics & Gynecology |
| $200,000 | $179,615 | $35,923 | 18% |  |
| $205,000 | $184,615 | $36,923 | 18% | Surgery |

^a^Discretionary income: earnings exceeding 150% of the 2022 poverty line: $13,590. ^b^The "Payment/Total Income" column represents the maximum recommended debt service ratio for the respective income tier (row). ^c^2021 salaries were within $500 of $100,000: modeled as $100,000.
