# Supplementary material for: Healthcare educational debt in the united states: unequal economic impact within interprofessional team members
Source: BMC Med Educ. 2023 Sep 14;23:666. doi: 10.1186/s12909-023-04634-1 (PMC10503048; doi:10.1186/s12909-023-04634-1)
Supplement: Supplementary file 2 — Supplementary Material 2 [file 12909_2023_4634_MOESM2_ESM.docx]

**Supplement 1. Data sources for estimates of undergraduate and graduate debt.**

| **Data Source Type** | **Profession** | **Data Source** | **Undergraduate Debt** | **Graduate Debt** |
| --- | --- | --- | --- | --- |
| Direct survey of students | Dentistry | 2021 American Dental Education Association | 16700 | 284900 |
| (highest reliability) | Medicine (all specialties) | 2021 Association of American Medical Colleges | 28000^c^ | 200000^c^ |
|  | Nurse Practitioner | 2017 American Association of Colleges of Nursing | 20566^a^ | 55000^e^ |
|  | Optometry | 2021 Association of Schools and Colleges of Optometry | 11324 | 147524 |
|  | Pharmacy | 2021 American Association of Colleges of Pharmacy | 20566^a^ | 173561 |
|  | Physical Therapy | Benchmarking in PT Education Study Waves 1-3 | 16804 | 82788 |
|  | Physician Assistant | 2019 Physician Assistant Education Association | 30000^d^ | 105000^d^ |
| Government database | Audiology | National Center for Educational Statistics: Other doctorate (non-Ph.D.) | 20566^a^ | 140349^f^ |
| (high reliability) | Genetic Counseling | National Center for Educational Statistics: Other master of science (M.S.) | 20566^a^ | 58415^f^ |
|  | Occupational Therapy | National Center for Educational Statistics: Other master of science (M.S.) | 20566^a^ | 58415^f^ |
| Educational foundation | Registered Nurse | The Institute for College Access and Success | 29096^b^ | N/A |
| (moderate reliability) | Bachelor's Degree | The Institute for College Access and Success | 29096^b^ | N/A |
| Other sources | Radiation Therapy | The Institute for College Access and Success, Cost of training program | 29096^b^ | 21436^g^ |
| (uncertain reliability) | Chiropractic | Extrapolated from peer-reviewed manuscript (Lorence et al 2014) | 20566^a^ | 115668^h^ |

a) No separate estimate of undergraduate debt available. Substituted the mean undergraduate debt for professions with direct survey data of students. b) Undergraduate debt estimated as mean undergraduate debt reported for 50 states and D.C. c) Source reports debt as median. d) Source excludes individuals with $0 debt: used median (rather than mean) to create greater congruence with other professions. e) Source reports debt as a range ($40k-$55k). Data are from 2017: used upper limit ($55k) to estimate 2021 debt. f) Source data are from 2016. Computed 2007-2016 CAGR from source data and extrapolated 2021 debt. g) No global debt estimate available. Estimated debt as cost of training program listed on institutional web pages. h) Source contains 2014 data. Computed graduate debt CAGR for other available professions (optometry, pharmacy, medicine, physician assistant: 2.1%) and extrapolated 2021 debt estimate.
